# Study on the Threshold of Serum Ferritin Required for Erythropoiesis and Iron Sufficiency in Hemodialysis Patients

**DOI:** 10.3390/ijms27041754

**Published:** 2026-02-12

**Authors:** Chie Ogawa, Ken Tsuchiya, Taku Morito, Naohisa Tomosugi, Kunimi Maeda

**Affiliations:** 1 Maeda Institute of Renal Research, Kawasaki 211-0063, Japan; morito@maeda-irr.com (T.M.); kuni@maeda-irr.com (K.M.); 2 Biomarker Society, Inc., Kawasaki 211-0063, Japan; tsuchiya@twmu.ac.jp (K.T.); tomosugi@kanazawa-med.ac.jp (N.T.); 3 Department of Nephrology, Tokyo Women’s Medical University, Tokyo 162-8666, Japan; 4 Division of Systems Bioscience for Drug Discovery Project Research Center, Medical Research Institute, Kanazawa Medical University, Kahoku 920-0293, Japan

**Keywords:** hemodialysis, ferritin, hemoglobin, oral iron replacement therapy

## Abstract

Serum ferritin (Ft) reflects total body iron stores and serves as a reference indicator for iron supplementation. However, optimal Ft level remains unclear in hemodialysis (HD) patients. We previously reported that total body iron (TBI), a novel index defined as the sum of red blood cell iron and storage iron, increases during oral iron replacement therapy (OIRT) and remains stable once iron sufficiency is achieved. In this study, we analyzed data from 100 OIRT courses in 79 maintenance HD patients. We examined the relationship between changes in hemoglobin (Hb) and Ft (⊿Hb, ⊿Ft) at 4 and 7 months after the initiation of OIRT, during which TBI remained stable, to determine the Ft level required for erythropoiesis. At 7 months, compared with 4 months, mean Hb significantly decreased by 0.2 g/dL (*p* = 0.03), while median Ft significantly increased from 60 to 75.0 ng/mL (*p* < 0.01). After adjustment for TBI, a significant inverse relationship was observed between ⊿Hb and ⊿Ft (β = −35.9, 95% CI −40.1 to −31.7, *p* < 0.001). These results indicate that an increase of 1 g/dL in Hb requires approximately 30–40 ng/mL of Ft, suggesting that this threshold may be useful for guiding iron supplementation in the treatment of anemia.

## 1. Introduction

Serum ferritin (Ft) levels are commonly used as an indicator of total body iron stores and serve as a basis for determining the need for iron supplementation therapy. However, Ft is influenced by factors other than iron status, including chronic inflammation, malignancy, and liver disease. Therefore, in patients with chronic kidney disease (CKD), who are often in a chronic inflammatory state, the optimal Ft level remains controversial, and Ft thresholds used for iron supplementation differ markedly between Western countries and Japan [1,2,3]. Since functional iron deficiency is also not uncommon, it is difficult to assess iron status based on Ft levels alone; therefore, evaluation of iron status is performed in combination with transferrin saturation (TSAT), an indicator of available iron.

Iron is an essential ion involved in vital biological processes; however, it also possesses toxic properties. Therefore, total body iron is tightly regulated within a semi-closed system, in which the very small daily losses of iron (approximately 1–2 mg/day) through desquamation of the intestinal mucosa, sweating, and other minor routes are compensated for by intestinal absorption. Once body iron stores are sufficient, a mucosal block mechanism is activated to prevent iron overload.

The main regulator of this system is hepcidin. When hepcidin binds to ferroportin, an iron transport protein expressed on the cell membranes of enterocytes, reticuloendothelial macrophages, and hepatocytes, ferroportin is degraded, resulting in reduced intestinal iron absorption and decreased iron release into the circulation from these cells. Because hepcidin expression is upregulated by iron and inflammatory signals, appropriate iron management requires not only the control of inflammation but also the maintenance of optimal body iron levels. To achieve this, investigating physiological iron dynamics is considered to be crucial.

Approximately 65–70% of the total body iron is contained within red blood cells (RBC) as hemoglobin (Hb), while most of the remaining iron is stored in ferritin and supplied as needed. Since these two compartments account for the majority of body iron, we previously proposed a new indicator, Total Body Iron (TBI)—defined as the sum of erythrocyte iron and storage iron—and used it to examine the physiological iron dynamics during oral iron replacement therapy (OIRT). In that study, we demonstrated that oral iron supplementation is effective even in patients undergoing hemodialysis (HD), and that once iron stores are replete, the total body iron level remains constant [4].

Under conditions where body iron stores are stable, the relationship between changes in Hb and Ft levels may provide insight into the Ft concentration required for erythropoiesis. Furthermore, Ft levels observed under iron-sufficient conditions may help elucidate the optimal range of Ft for appropriate iron management. Therefore, in the present study, we analyzed data obtained during OIRT to investigate the relationship between Hb and Ft under TBI stability. Based on this relationship, we aimed to identify Ft levels that can serve as indicators for iron supplementation during anemia management, as well as those that represent iron sufficiency.

In addition, recent studies have demonstrated that the severity of TSAT abnormalities is associated not only with anemia but also with prognosis and cardiovascular diseases [5,6,7,8]. Therefore, by examining the relationship between body iron stores, Ft levels, and TSAT, we also investigated whether the control of TSAT is artificially achievable.

Furthermore, we investigated the associations of hepcidin with Ft, TSAT, and mean corpuscular volume (MCV) under iron-replete conditions.

## 2. Results

### 2.1. Patient Characteristics

The median age of the patients was 70.7 (57.2–77.8) years, with a median dialysis duration of 7.5 (2.4–32.4) years. Men accounted for 74 courses, and 33 had diabetic nephropathy. At OIRT initiation, mean Hb was 10.4 ± 0.7 g/dL, red blood cells (RBC) 345.7 ± 36 × 10^4^/μL, mean corpuscular hemoglobin (MCH) 30.3 ± 2.2 pg, median Ft 24.9 (18.8–33.8) ng/mL, and mean TSAT 18.2 ± 6.1%. The median weekly Darbepoetin alfa (DA) dose was 20 (10–30) μg (Table 1).

### 2.2. Changes in TBI During OIRT

**Figure 1:**
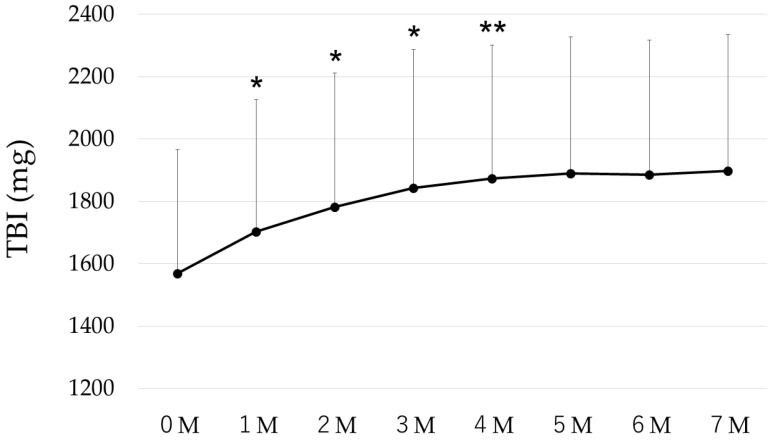
Changes over time in TBI, *; *p* < 0.01, **; *p* < 0.05. TBI increased significantly compared to the previous month up to 4 M, and then stabilized.

Following the initiation of OIRT, TBI showed a significant increase compared with the preceding month until month 4 (4 M), after which it reached a plateau. (Figure 1).

### 2.3. Comparison of TBI, Erythrocyte- and Iron-Related Parameters, and Darbepoetin α Doses at 0, 4, and 7 Months

**Figure 2:**
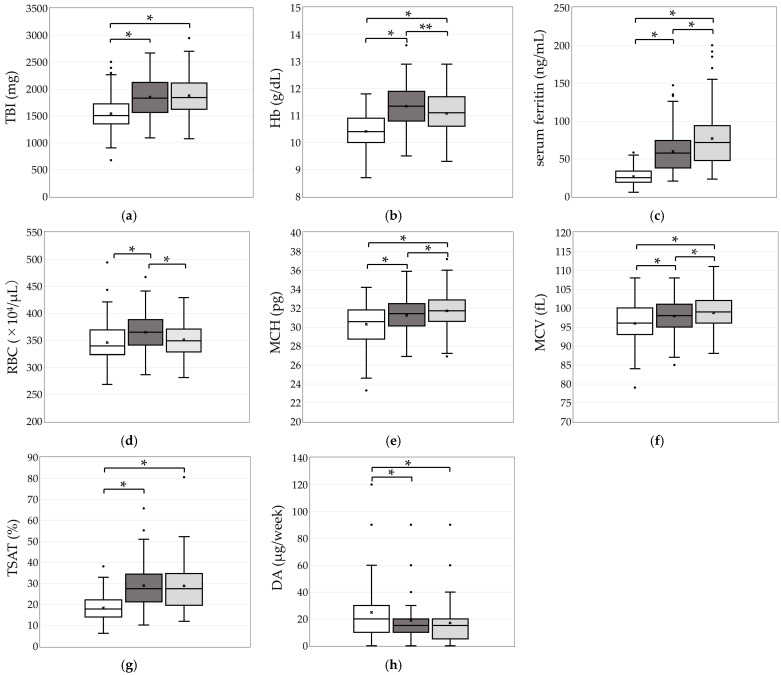
Comparison of TBI, RBC indices, iron-related indexes, and darbepoetin α doses at 0, 4, and 7 M (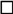, 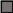, 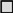). 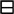; median (interquartile range), ×; mean, *; *p* < 0.01, **; *p* < 0.05. (**a**) Total Body Iron; (**b**) Hemoglobin; (**c**) serum ferritin; (**d**) Red blood cell count; (**e**) mean corpuscular hemoglobin; (**f**) mean corpuscular volume; (**g**) Transferrin saturation; (**h**) Darbepoetin α doses. Between 4 and 7 months, TBI remained stable, whereas Hb significantly decreased and Ft significantly increased. In contrast, TSAT remained unchanged. Following the reduction in DA, RBC counts decreased at 7 months, while MCH and MCV increased.

TBI levels at 4 and 7 M after the initiation of OIRT were nearly identical (Figure 2a). The mean Hb level (g/dL) showed a significant increase at 4 and 7 M (11.4 ± 0.8 and 11.1 ± 0.8, respectively) compared with the baseline value at 0 M (10.4 ± 0.7) (*p* < 0.01). However, at 7 M, the Hb level was significantly lower than that at 4 M (*p* = 0.03, Figure 2b). The median Ft increased continuously and significantly from 0 to 4 M (57.3, IQR: 37.6–74.2) and further to 7 M (71.3, IQR: 47.5–93.8) (both *p* < 0.01, Figure 2c). MCH and MCV also increased significantly up to 7 M (*p* < 0.01, Figure 2e,f). The mean TSAT (%) rose significantly from 0 M to 4 M (28.9 ± 11.1%), but remained nearly unchanged at 7 M (28.8 ± 11.7%, Figure 2g). The median Darbepoetin α (DA) dose decreased significantly to 15 (10–20) µg at 4 M and remained stable thereafter (Figure 2h).

### 2.4. Relationships Between Changes in Hb and Ft from 4 to 7 M (⊿Hb, ⊿Ft), and Between Ft at 7 M and ⊿Hb

**Figure 3:**
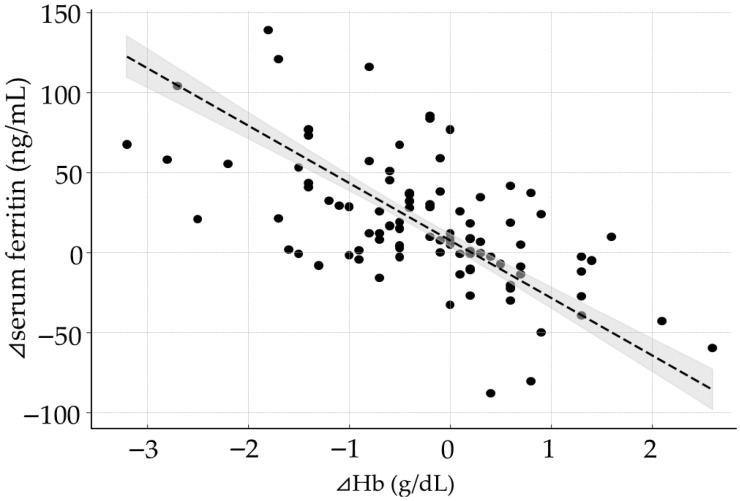
Relationship Between ⊿Hb and ⊿Ft. A significant positive correlation was observed between changes in Hb (⊿Hb) and changes in Ft (⊿Ft). The dotted line represents the fitted linear regression (⊿Ft = 1.9 − 35.9 × ⊿Hb + 0.2 × ⊿TBI, ⊿TBI was fixed at the mean value. R^2^ = 0.76, *p* < 0.001). The gray area indicates the 95% confidence interval. Each dot represents an individual subjec.

A significant negative correlation was observed between ⊿Hb and ⊿Ft (*p* < 0.001, Figure 3). In the multivariate analysis for ⊿Ft adjusted for the changes in TBI (⊿TBI), the regression coefficient for ⊿Hb was –35.9 (95% confidence interval: −40.1 to −31.7, *p* < 0.001) (Table 2). Furthermore, In the multivariate analysis examining the relationships among Ft at 7 M, ⊿Hb, and ⊿TBI, the intercept was 67.8 (60.7–75.0) ng/mL for Ft at 7 M.

### 2.5. Relationship Between Changes in RBC, MCH from 4 M to 7 M (⊿RBC, ⊿MCH), and ⊿Ft

**Figure 4:**
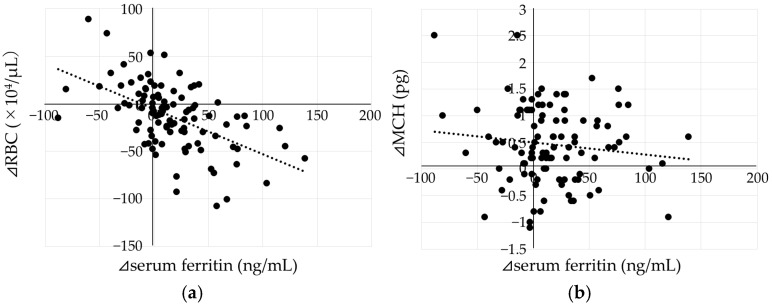
Relationship between ⊿RBC (**a**), ⊿MCH (**b**) and ⊿Ft. ⊿Ft showed a significant negative correlation with ⊿RBC (⊿Ft = −0.48 × ⊿RBC − 5.42, R^2^ = 0.29, *p* < 0.001), but it was not correlated with ⊿MCH. Each dot represents an individual subject, and the dotted line indicates the linear regression line.

A significant negative correlation was observed between ⊿RBC and ⊿Ft (*p* < 0.001, Figure 4a). In contrast, no significant correlation was found between ⊿MCH and ⊿Ft (*p* = 0.23, Figure 4b).

### 2.6. Relationship Between Ft and TSAT

**Figure 5:**
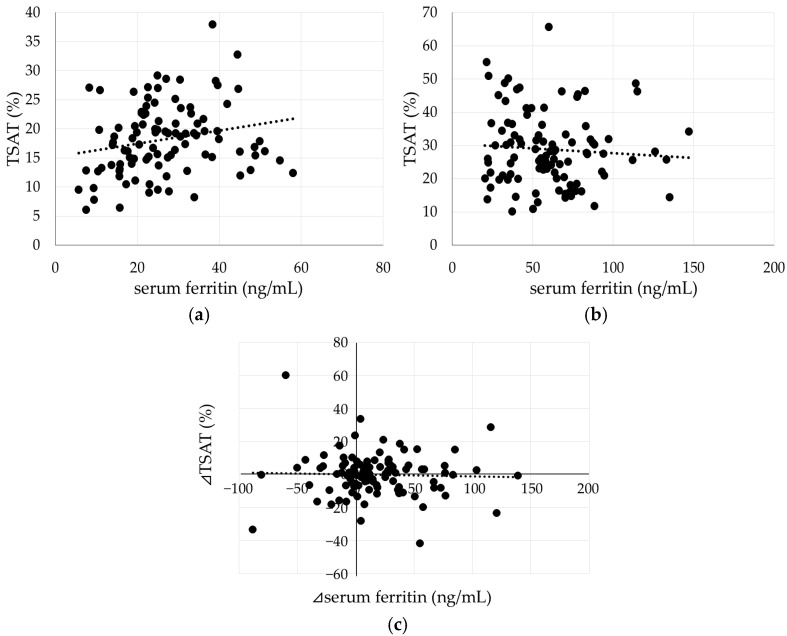
Relationship between Ft and TSAT at 0 M (**a**), and 4 M (**b**), and ⊿TSAT and ⊿FT (**c**). A significant positive correlation between Ft and TSAT was observed at 4 M (TSAT = 0.11 × Ft + 15.17, R^2^ = 0.05, *p* = 0.03); however, this correlation was no longer present at 7 M. No correlation was observed between the changes in Ft and TSAT from 4 to 7 months. Each dot represents an individual subject, and the dotted line indicates the linear regression line.

At 0 M, when Ft levels were low and consistent with absolute iron deficiency, Ft and TSAT showed a significant positive correlation (*p* = 0.03, Figure 5a). However, at 4 M, when body iron stores were replete, the correlation between Ft and TSAT disappeared (*p* = 0.49, Figure 5b). Furthermore, no correlation was observed between changes in Ft and TSAT from 4 M to 7 M (*p* = 0.73). In many cases, TSAT fluctuated within a range of ±10% even when TBI was in a stable state (Figure 5c).

### 2.7. Relationships Among Hepcidin, Ferritin, TSAT, and MCV Under Iron-Replete Conditions

In the present study, the median TBI/steady-state TBI ratio of the data used for the analysis of hepcidin was 1.01 (interquartile range, 0.98–1.06).

**Figure 6:**
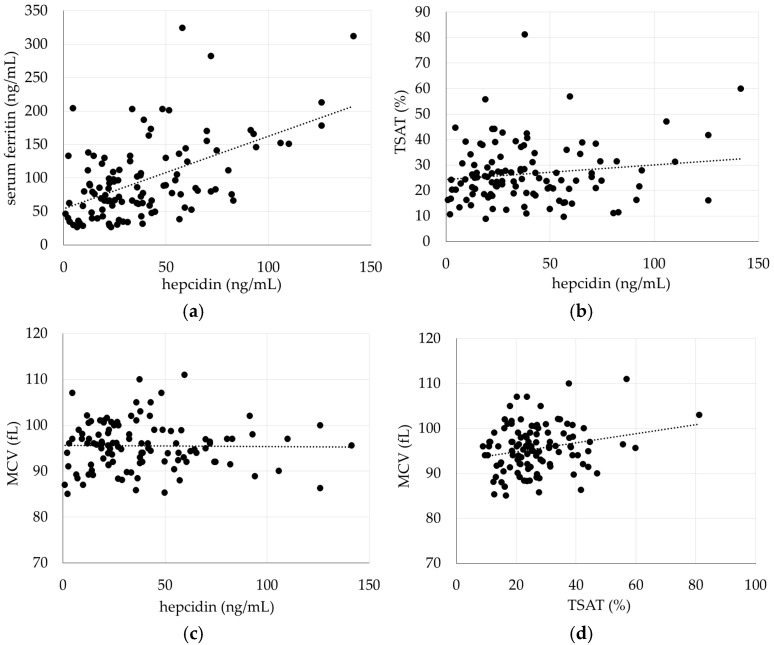
Relationship among hepcidin, Ft, TSAT and MCV. Relationship between hepcidin and Ft (**a**), and TSAT (**b**), and MCV (**c**). Relationship between MCV and TSAT (**d**). Hepcidin showed a positive correlation with Ft (Ft = 1.09 × hepcidin + 53.83, R^2^ = 0.29), but no significant correlation with TSAT or MCV. MCV showed a positive correlation with TSAT (MCV = 0.10 × TSAT + 92.81, R^2^ = 0.05). Each dot represents an individual subject, and the dotted line indicates the linear regression line.

The median hepcidin level was 33.1 (19.0–55.2) ng/mL and showed a strong positive correlation with serum ferritin (r = 0.54, *p* < 0.001, Figure 6a). In contrast, no significant correlation was observed between hepcidin and TSAT or MCV (*p* = 0.14 and *p* = 0.88, respectively, Figure 6b,c). MCV showed a weak correlation with TSAT (r = 0.23, *p* = 0.02, Figure 6d).

## 3. Discussion

Iron metabolism functions as a semi-closed circuit, in which most of the iron required for erythropoiesis is supplied through the recirculation of existing body iron. In healthy individuals, because erythropoiesis remains constant, Ft levels also remain stable when iron stores are adequate [9,10]. In contrast, in patients undergoing HD, erythropoiesis is artificially regulated by erythropoiesis-stimulating agents (ESAs) or hypoxia-inducible factor prolyl hydroxylase (HIF-PH) inhibitors. Consequently, Hb levels fluctuated, and Ft concentrations may vary even when body iron stores are stable.

In the present study, dose reduction of DA was implemented to improve anemia during OIRT, which resulted in a decrease in RBC count at 7 M, accompanied by a corresponding increase in Ft levels. The findings indicated that an increase of 1 g/dL in Hb corresponds to an iron requirement equivalent to approximately 30–40 ng/mL of Ft, when adjusted for changes in TBI.

On the other hand, regarding Ft level under an iron-sufficient condition, at 4 M the subjects may be in the early phase of repletion, and some cases might not yet have achieved full iron sufficiency. Therefore, using the relationship between Ft and ⊿Hb at 7 M—when the body iron stores are considered to be in a more stable state—we examined the predicted Ft at 7 M under the assumption that no iron shift occurs due to fluctuations in Hb. The results suggested that the predicted value would likely fall within the range of approximately 60.7–75.0 ng/mL. This value was very close to that reported in studies using isotopes to examine iron absorption in HD patients, in which iron absorption was nearly zero at Ft level of approximately 60 ng/mL, as well as to the WHO threshold of Ft < 70 ng/mL for defining iron deficiency in the presence of chronic inflammation [11,12].

These observations are consistent with previous findings from studies in Japanese HD patients. In an observational study, a positive correlation between Hb and Ft was observed in the group with Ft levels below 50 ng/mL, whereas no such correlation was found in patients with higher Ft levels [13]. Moreover, an analysis using the nationwide database of the Japanese Society for Dialysis Therapy reported that, although minor variations were observed depending on the type of ESA used, the highest Hb levels were achieved at Ft concentrations between 40 and 80 ng/mL [14]. Similarly, another report investigating OIRT demonstrated that Hb levels peaked when Ft was approximately 40 ng/mL, and that Ft increased as Hb declined [15,16]. Although a Ft level below 100 ng/mL is often used as a criterion for iron deficiency [17,18,19], previous reports as well as our present findings suggest that, in HD patients, iron stores sufficient to sustain erythropoiesis may be maintained even at lower Ft levels, provided that no fluctuations in Hb occur.

Regarding Ft, it should be acknowledged as a limitation that Ft levels may not accurately reflect total body iron stores under conditions of iron overload or active inflammation. However, within the physiological range, Ft remains the biomarker that most closely reflects body iron stores and is widely used for the diagnosis of absolute iron deficiency in current clinical guidelines [20,21,22]. Although the assessment of Ft in HD patients with chronic inflammatory conditions warrants caution, the Ft thresholds proposed by the WHO were established with consideration of inflammatory markers such as C-reactive protein (CRP) and α1-acid glycoprotein (AGP) [12]. In addition, previous studies have shown that the relationship between hepcidin and Ft does not differ significantly between healthy individuals and HD patients [23]. A recent study evaluating iron absorption during ferric citrate therapy demonstrated that combined changes in Hb and Ft levels could be used to infer changes in total body iron. In that study, patients with baseline mean Ft levels of approximately 70–80 ng/mL showed heterogeneous changes in total body iron, whereas total body iron remained stable in patients with baseline mean Ft levels of approximately 120–140 ng/mL [24]. These findings, which are close to the Ft range identified in the present study, suggest that a physiological mucosal block may be operative at these Ft levels. Taken together, Ft thresholds identified in the present study are considered to be clinically useful.

Based on these findings, when Hb levels are maintained, body iron stores are likely to be sufficient at Ft levels of approximately 60–75 ng/mL. However, even within the same ferritin range (60–75 ng/mL), attempts to increase Hb levels would require an additional Ft increase of approximately 30–40 ng/mL per 1 g/dL of Hb, indicating the need for iron supplementation. Therefore, the requirement for iron supplementation depends not only on Ft levels but also on whether enhanced erythropoiesis is required.

In the JSDT guidelines, iron supplementation is indicated when the target Hb level cannot be maintained, and a serum ferritin level below 100 ng/mL has been used as the threshold [3]. When the requirement for Hb elevation is taken into account, our findings appear to be consistent with these guideline recommendations. In contrast, the 2026 KDIGO anemia guidelines propose higher ferritin thresholds for defining iron deficiency in HD patients (ferritin ≤ 500 ng/mL and TSAT ≤ 30%) and recommend intravenous iron supplementation in the presence of anemia [1]. Although these values were proposed based on considerations such as prognosis, cardiovascular risk, and ESA dose reduction, they may be excessively high when interpreted as thresholds for adequate total body iron stores.

Regarding TSAT, in the present study, TSAT showed a positive correlation with Ft under conditions of absolute iron deficiency. TSAT increased with the improvement of absolute iron deficiency; however, once body iron stores reached a sufficient level, TSAT was no longer influenced by Ft. It was suggested that even when Ft increases under iron-sufficient conditions, it does not lead to an enhanced supply of bioavailable iron, but rather depends on the individual’s intrinsic efficiency of iron utilization. Furthermore, TSAT occasionally decreased even in HD patients with sufficient body iron stores, suggesting that it may not be a suitable indicator for iron supplementation.

To investigate iron metabolism in greater detail under iron-replete conditions, we examined the relationships between hepcidin, iron-related parameters, and indices of erythropoiesis.

Because this was a retrospective observational study, hepcidin was not measured contemporaneously with other laboratory parameters. Therefore, we utilized data from a previously established database in which hepcidin measurements had been performed and selected cases presumed to be in an iron-replete state.

The ratio of total body iron (TBI) to the predicted iron-replete value (steady-state TBI) had a median of 1.01 (0.98–1.06), suggesting that this cohort closely resembles a population that has achieved iron repletion through oral iron supplementation.

The correlation between hepcidin, a major regulator of iron metabolism, and Ft has been reported previously. Under normal conditions, because erythropoiesis is relatively stable, Ft is generally considered to reflect total body iron stores. However, in HD patients, erythropoiesis is not constant, and Ft does not necessarily represent total body iron. The present analysis suggests that hepcidin is associated with iron storage rather than total body iron content. Because the mucosal block does not operate with intravenous iron administration, iron supplementation can be readily achieved even after total body iron stores are replete, potentially leading to excessive accumulation of storage iron. Given that elevated hepcidin levels raise concerns regarding impaired iron utilization, our findings suggest that iron supplementation strategies should be carefully managed to avoid excessive increases in ferritin levels.

Because hepcidin induces the degradation of the iron transporter ferroportin, lower hepcidin levels would be expected to facilitate iron export from cells into the circulation when body iron stores are sufficient. However, in the present study, no association was observed between hepcidin and either the supply of bioavailable iron or iron utilization in erythropoiesis. In contrast, a correlation was observed between TSAT and MCV, indicating that an adequate supply of bioavailable iron is essential for iron utilization during erythropoiesis. These results suggest that, when body iron stores are adequate, circulating iron supply and erythropoietic iron utilization may be influenced more strongly by factors other than hepcidin. Further studies are required to clarify the regulatory mechanisms of iron metabolism.

This study has several limitations. First, because it was a retrospective and observational study with a relatively small number of enrolled patients, the possibility that unrecognized confounding factors remain cannot be excluded. Second, although a target Hb level was predefined, anemia management was performed at the discretion of each attending physician, which may have introduced variability in treatment. Although the median CRP level was low (0.09 ng/mL), suggesting good inflammatory control in this cohort, we cannot entirely rule out the potential influence of chronic inflammation on serum ferritin levels.

In the future, a large-scale prospective study is warranted to further evaluate the effects of oral iron therapy on Hb levels and iron status in patients undergoing HD.

## 4. Materials and Methods

### 4.1. Patients

This study included 79 outpatients undergoing maintenance HD at our hospital who received low-dose OIRT between January 2018 and April 2022. All patients underwent three sessions of maintenance HD per week, each lasting 3 to 5 h, for at least 3 months prior to enrollment.

Written informed consent for data collection and analysis was obtained from all participants. The study protocol was approved by the Ethics Committee of the Biomarker Society, Inc., which consists of five committee members, including external experts.

### 4.2. Methods

This retrospective study analyzed data from 100 courses of OIRT administered to 79 maintenance HD outpatients at our hospital. OIRT was initiated in patients with Ft levels below 60 ng/mL and Hb levels below 12 g/dL. Patients received 50–120 mg of elemental iron daily, administered as either ferrous citrate (50 or 100 mg/day, Eisai Co., Ltd., Tokyo, Japan) or ferric citrate (250 or 500 mg/day, corresponding to 60 or 120 mg of elemental iron, respectively, Torii Pharmaceutical Co., Ltd., Tokyo, Japan). Blood samples were collected at the first dialysis session of each week. Anemia-related parameters were measured twice a month, and the dose of DA (Kyowa Kirin Co., Ltd., Tokyo, Japan) a long-acting ESA, was adjusted to maintain a target Hb level of 10–12 g/dL in accordance with the Japanese Society for Dialysis Therapy (JSDT) guidelines. Iron-related indices were measured once a month. TSAT was calculated using the formula: TSAT = Fe/TIBC × 100.

In cases where multiple OIRT courses were administered to the same patient, data from the initiation of each course were used for analysis.

TBI is a novel indicator for estimating in vivo iron levels, proposed by Cable et al. [25]. Most of the body’s iron is accounted for by RBC iron (the iron contained in RBC Hb) and iron stores, so the sum of these parameters is considered to represent the approximate amount of iron in vivo. The amount of iron in 1 g of Hb is assumed to be 3.4 mg and the estimated blood volume (EBV) was calculated using Nadler’s formula, which utilizes height, weight, and sex, as follows.

TBI (mg) = RBC iron + iron stores
RBC iron (mg) = 3.4 mg × Hb value (g/dL) × 10 × EBV (L) × 0.91
Iron stores (mg) = (−13.8588 + 0.3929 × G + 15.5999log10(F) − 2.0519(log10(F))2) × body weight (kg)
F: ferritin (ng/mL); G: men = 0, women = 1.

TBI significantly increased after the initiation of OIRT and then plateaued from 4 M onward. Therefore, we calculated the changes in Hb and Ft levels (⊿Hb and ⊿Ft, respectively) between 4 M and 7 M, when patients were considered to be in an iron-sufficient state, in order to investigate the Ft level required for erythropoiesis. In addition, the relationships among changes in erythrocyte- and iron-related parameters, and darbepoetin α doses were analyzed to examine the physiological dynamics of iron metabolism.

To investigate the effects of hepcidin under iron-replete conditions, we analyzed data from September 2008 and April 2013, when serum hepcidin levels were measured. A total of 110 subjects satisfying TBI/steady-state TBI ≥ 0.95 were included, and the relationships among hepcidin and Ft, TSAT, and MCV, an indicator of iron utilization in erythropoiesis, were examined. Hepcidin was measured using a quantitative method involving liquid chromatography coupled with tandem mass spectrometry [26]. Steady-state TBI is a value defined in our previous study, representing the TBI at which body iron sufficiency is achieved through oral iron supplementation, and is predicted by the following equation: steady-state TBI = −791.914 + 1628.606 × BSA (Target Hb level; 10–12 g/dL, R^2^ = 0.883) [4].

### 4.3. Statistical Analysis

Data are expressed as the mean ± standard deviation (SD) or as the median with interquartile range (IQR). Data normality was assessed using the Kolmogorov–Smirnov test, and appropriate parametric or nonparametric tests were applied accordingly. For comparisons of the values of each parameter at 0, 4, and 7 M, a one-way repeated-measures analysis of variance was performed for normally distributed variables, whereas the Friedman test was applied for non-normally distributed variables. Bonferroni’s multiple comparison test was conducted as a post hoc analysis. The correlations among the variables were examined using Pearson’s product-moment correlation coefficient. To adjust the relationship between ⊿Ft and ⊿Hb for ⊿TBI, multivariate linear regression analysis was performed.

All statistical analyses were two-tailed, and *p* < 0.05 was considered statistically significant. Analyses were performed with EZR (Jichi Medical University, Tochigi, Japan).

## 5. Conclusions

This study suggested that, in patients undergoing HD, approximately 30–40 ng/mL of Ft may be utilized for each 1 g/dL increase in Hb, and that body iron stores may be sufficient at Ft levels of approximately 60–75 ng/mL under stable anemia management without Hb fluctuation. Furthermore, our findings indicate that not only total body iron but also iron availability is tightly regulated in the body, and that the supply of bioavailable iron cannot be fully explained by hepcidin alone, suggesting the need for further investigation. In hemodialysis (HD) patients, evaluating iron sufficiency and the need for iron supplementation based solely on serum ferritin levels is difficult; therefore, it is important to assess these parameters in conjunction with hemoglobin (Hb) control status.

## Figures and Tables

**Table 1:** Patient characteristics at baseline.

Variables	
N (courses)	100
Age (years)	70.7 (57.2–77.8)
Sex; men (*n*)	74
HD vintage	7.5 (2.4–32.4)
Diabetes nephropathy (*n*)	33
Hb (g/dL)	10.4 ± 0.7
RBC (×10^4^/μL)	345.7 ± 36.0
MCH (pg)	30.3 ± 2.2
MCV (fL)	96.0 ± 5.6
s-Fe (μg/dL)	51.2 ± 17.5
TIBC (μg/dL)	284.9 ± 41.0
TSAT (%)	18.2 ± 6.1
serum ferritin (ng/mL)	24.9 (18.8–33.8)
Darbepoetin α (μg/week)	20 (10–30)
C-reactive protein (mg/dL)	0.09 (0.01–5.99)

Values are shown as the number, mean ± standard deviation, or median (IQR; interquartile range). Hb; Hemoglobin, RBC; Red blood cells, MCH; mean corpuscular hemoglobin, MCV; mean corpuscular volume, TIBC; total iron-binding capacity, TSAT; Transferrin saturation.

**Table 2:** Multivariable regression analysis of the change in Ft (ng/mL) from 4 to 7 months.

Variables	β	95% Confidence Interval	*p* Value
⊿Hb	−35.9	(−40.1 to −31.7)	<0.001
⊿TBI	0.2	(0.2 to 0.3)	<0.001

⊿Hb; (per 1 g/dL increase), ⊿TBI; (per 1 mg increase).

## Data Availability

The original contributions presented in this study are included in the article. Further inquiries can be directed to the corresponding authors.

## Abbreviations

The following abbreviations are used in this manuscript:

Ft	Serum ferritin
HD	hemodialysis
TBI	total body iron
Hb	hemoglobin
OIRT	oral iron replacement therapy
CKD	chronic kidney disease
TSAT	transferrin saturation
RBC	red blood cells
MCH	mean corpuscular hemoglobin
DA	Darbepoetin alfa
ESAs	erythropoiesis-stimulating agents
HIF-PH	hypoxia-inducible factor prolyl hydroxylase
JSDT	Japanese Society for Dialysis Therapy
EBV	estimated blood volume
